# Chronic Nicotine Exposure Increases Hematoma Expansion following Collagenase-Induced Intracerebral Hemorrhage in Rats

**DOI:** 10.3390/biom12050621

**Published:** 2022-04-21

**Authors:** Ashish K. Rehni, Sunjoo Cho, Zhexuan Zhang, Weizhao Zhao, Ami P. Raval, Miguel A. Perez-Pinzon, Kunjan R. Dave

**Affiliations:** 1 Peritz Scheinberg Cerebral Vascular Disease Research Laboratory, Department of Neurology, Leonard M. Miller School of Medicine, University of Miami, Neurology Research Building, 1420 NW 9th Avenue, Room # 203, Miami, FL 33136, USA; axr539@miami.edu (A.K.R.); s.cho6@med.miami.edu (S.C.); araval@med.miami.edu (A.P.R.); perezpinzon@med.miami.edu (M.A.P.-P.); 2 Department of Neurology, University of Miami Miller School of Medicine, Miami, FL 33136, USA; 3 Department of Biomedical Engineering, University of Miami, Coral Gables, FL 33146, USA; z.zhang3@miami.edu (Z.Z.); wzhao@med.miami.edu (W.Z.); 4 Neuroscience Program, University of Miami Miller School of Medicine, Miami, FL 33136, USA

**Keywords:** cigarette, tobacco, smoking, risk factor, stroke

## Abstract

Spontaneous intracerebral hemorrhage (sICH) is a deadly stroke subtype, and tobacco use increases sICH risk. However epidemiological studies show that, there are no confirmatory studies showing the effect of tobacco use on sICH outcome. Therefore, we evaluated the effect of chronic nicotine exposure (as a surrogate for tobacco use) on outcomes following sICH. Young male and female rats were randomly assigned to either nicotine (4.5 mg/kg b.w. per day) or vehicle (saline) treatment (2–3 weeks) groups. sICH was induced by injecting collagenase into the right striatum. Neurological score and hematoma volume were determined 24 h post-sICH. The hematoma volumes in nicotine-treated male and female rats were significantly higher by 42% and 48% when compared to vehicle-treated male and female rats, respectively. Neurological deficits measured in terms of neurological score for the nicotine-treated male and female groups were significantly higher when compared to the respective vehicle-treated male and female groups. Our results show that chronic nicotine exposure increases hematoma volume post-sICH in rats of both sexes. Identifying the mechanism of nicotine-dependent increase in hematoma growth post-sICH will be crucial to understanding the detrimental effect of tobacco use on the severity of bleeding following intracerebral hemorrhage.

## 1. Introduction

As defined by the American Heart Association, intracerebral hemorrhage is “a focal collection of blood within the brain parenchyma or ventricular system that is not caused by trauma” [1]. Spontaneous intracerebral hemorrhage (sICH) is a serious form of stroke that accounts for as high as 28% of all strokes worldwide and is associated with substantial morbidity and mortality [2,3,4,5]. In 2019, sICH was responsible for 2.9 million deaths worldwide [2]. According to one systematic review, the rate of sICH incidence in different populations varies from 20 to 50 per 100,000 person-years [6]. sICH has a one-month mortality of approximately 40%, and only 10-40% of survivors lead an independent life [6,7]. There are many modifiable risk factors that predispose subjects to sICH. These include high blood pressure, increased alcohol consumption, smoking, reduced triglyceride, and low-density lipoprotein levels [8,9]. However, non-modifiable risk factors for sICH include kidney disease, sex, age, cerebral microbleeds, ethnicity, and cerebral amyloid angiopathy [8,9]. 

More than 480,000 people die because of active cigarette smoking, and approximately 41,000 people die of passive smoking every year in the USA [10]. Tobacco use is one of the major modifiable risk factors for sICH in both male and female subjects [11,12]. Cigarette smoking is a risk factor for sICH among subjects younger than 55 years of age, and its incidence increases with increasing consumption of cigarettes [13]. Tobacco use further enhances the propensity for hypertension or pregnancy-related sICH [14,15]. Cigarette smoking also enhances hematoma volume and mortality and worsens outcomes following sICH [16,17].

However, given the lack of scientific literature on the effect of tobacco use on sICH, there is a need to systematically study the effect of tobacco use on sICH using well-characterized animal models of the disease condition. Nicotine is one of the principal active constituents in tobacco, which is considered to produce detrimental effects on the cardiovascular system, as discussed previously [18]. Therefore, in this first study, we evaluated the effect of chronic nicotine exposure, a surrogate of tobacco use, on outcomes following sICH. 

## 2. Materials and Methods

### 2.1. Animals

Animal experiments were carried out per the guidelines stated in the Guide for the Care and Use of Laboratory Animals by the National Institutes of Health. The experimental procedures used were approved by the University of Miami Institutional Animal Care and Use Committee. We procured young male and female Sprague Dawley rats from Charles River Laboratories (Wilmington, MA, USA). Animals were randomly assigned to treatment groups, animals were excluded based on predefined measures of exclusion, and analysis was performed by blinded investigators. 

### 2.2. Nicotine Administration

Osmotic pumps (Alzet osmotic pump model number: 2ML2) (DURECT Corporation, Cupertino, CA, USA) were subcutaneously implanted into the rats, infusing either normal saline (vehicle) or nicotine bis-L-(+)-tartrate dihydrate (4.5 mg/kg b.w. per day) for 17 ± 1 days to mimic chronic nicotine exposure. The nicotine dose and route of administration chosen were aimed to maintain plasma nicotine and cotinine levels like those observed in chronic moderate smokers [19,20,21]. An earlier study confirmed the presence of nicotine in the brain when rats were treated using the dose and route described above [22]. 

### 2.3. Determination of the Stage of the Estrous Cycle

Earlier epidemiological studies identified sex-related differences in outcomes following sICH in patients [23,24]. Therefore, we evaluated the effect of chronic nicotine exposure on hematoma growth following collagenase-induced sICH in female rats. Estrous cycle monitoring was performed as described earlier [25]. In brief, vaginal smears were collected prior to sICH induction, and the identification of the estrous stage was conducted by microscopic examination of the smears. To avoid the influence of estrous cycle-related hormonal fluctuations on hematoma growth, sICH induction in female rats was performed during the diestrus stage.

### 2.4. sICH Induction

Body weight and blood glucose levels were measured prior to surgery. Rats were anesthetized using isoflurane in a mixture containing oxygen and nitrous oxide (30/70 ratio). The femoral vein was cannulated to allow for the administration of rocuronium and saline, and the femoral artery was cannulated to allow for blood sampling and blood pressure determination. The rats were then paralyzed with rocuronium and placed on a mechanical ventilator. Physiological parameters were monitored before and after collagenase injection and maintained within a normal range. The parameters included head and body temperature, blood pH, partial pressure of carbon dioxide (pCO_2_) and oxygen (pO_2_) in the blood, and mean arterial blood pressure (MABP). The rats were positioned in a stereotaxic frame, and a sagittal incision of approximately 2 cm was made in the skin. The skull was cleared of tissue to allow marking of the bregma. A burr hole of about 2 to 3 mm diameter was made on the right side of the skull using a manual drill. Coordinates of the burr hole were 3.2 mm medio-lateral and 0.2 mm antero-posterior of the bregma. The dura was gently opened through the burr hole using a needle without damaging the underlying brain tissue. Collagenase was injected into the right striatum using a Hamilton syringe (10 μL capacity, Hamilton Company, Reno, NV, USA) installed above the burr hole with a drill and injection robot (Neurostar, Tubingen, Germany) [26,27]. Briefly, the tip of the 30-G needle of the syringe containing the collagenase was inserted at a rate of 0.20 mm/s, 3.2 mm to the right, 6.5 mm deep, and 0.2 mm anterior to the bregma, and 0.12 U of collagenase dissolved in normal saline was injected in the brain (2.0 μL at a rate of 0.4 μL/min). The needle was left in place for an additional 5 min to allow diffusion of the solution into the brain tissue and avoid mechanical trauma and exodus of the injected fluid through the hole in the brain. Bone wax was used to seal the burr hole after removing the syringe. The skin was sutured back, and appropriate post-operative care was provided to the rat. MABP was recorded continuously during the surgery and was averaged every 10 min, and the values of three consecutive segments were averaged to compute the mean MABP for a 30 min period (Figure 4B,C).

### 2.5. Neurological Score Assessment

The neurological score was used to determine neurological impairment as described before [28,29]. This test was performed 24 h after sICH induction. This scale includes postural reflex, visual placing, tactile placing, and proprioceptive placing tests. A score of 0 and 12 indicated no and maximum impairment, respectively. 

### 2.6. Animal Perfusion and Brain Isolation

Rats were anesthetized ~24 h after collagenase injection using isoflurane in a mixture containing oxygen and nitrous oxide (30/70 ratio). This was followed by a median sternotomy, and an incision was made in the apex of the left ventricle of the heart. Polyethylene tubing (PE-240) was gently inserted into the aorta via the incised heart and fastened in place using a suture. An opening was made in the right auricle to facilitate exodus of the perfusate. Cold saline was then allowed to perfuse the rat body under a pressure of ~120 mmHg until clear exudate was seen. Rat brains were then harvested.

### 2.7. Quantification of Hematoma Volume

The brain was sectioned using single-edge industrial razor blades into 2 mm thick coronal slices. The rostral facet of the sections was defined as Side A, and the caudal facet of the sections was defined as Side B. The approximate bregma levels represented by Side A were 6.0, 4.0, 2.0, 0.0, −2.0, −4.0, and −6.0 mm. The approximate bregma levels represented by Side B were 4.0, 2.0, 0.0, −2.0, −4.0, −6.0, and −8.0 mm. The slices were then scanned at a resolution of 2400 dpi and 24-bit true color per pixel. Before analyzing, the images were checked for the presence of any artifacts. The hematoma area on either side was quantified using ImageJ software by an investigator blinded to treatment conditions. The mean hematoma values computed from both sides were used to determine the hematoma volume [27,30].

Individual sections from each scan were further processed. The hematoma area was identified as described earlier [27]. Computer-assisted image mapping for each of the treatment groups was used for hematoma frequency distribution analysis. Every section of the brain was then mapped into a corresponding digital rat brain atlas. Two disparate intensity values were given to regions identified as hematoma and normal brain tissue. The hematoma frequency distribution maps for the study groups were generated from Side A of the scans based on the occurrence of the hematoma on a pixel basis [28]. The mapped binary sections, between groups, were compared at 7 corresponding levels of the brain using Fisher’s exact test. The final images obtained after the processing are the mirror images of the originally scanned sections and show the hematoma in frequency maps to be on the opposite side of the brain. 

### 2.8. Experimental Protocol

**Figure 1:**
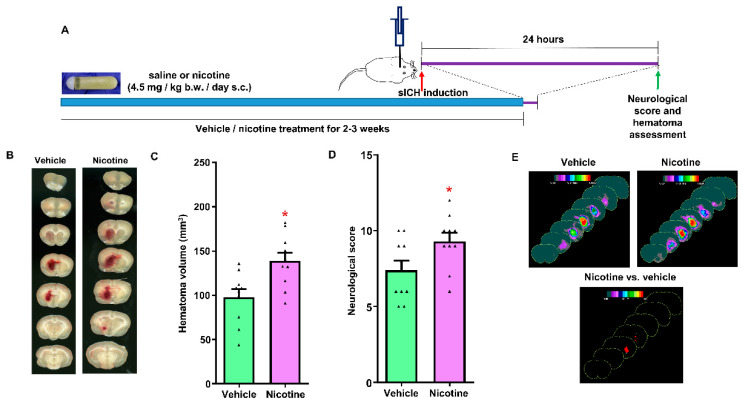
The effect of chronic nicotine treatment on hematoma expansion following collagenase-induced spontaneous intracerebral hemorrhage (sICH) in male rats: (**A**) experimental design; (**B**) representative example images showing hematoma volume following sICH; (**C**) hematoma volume; (**D**) neurological impairment; and (**E**) hematoma frequency maps at 7 coronal levels (bregma +6 mm to −6 mm). * *p* < 0.05 vs. vehicle group.

**Figure 2:**
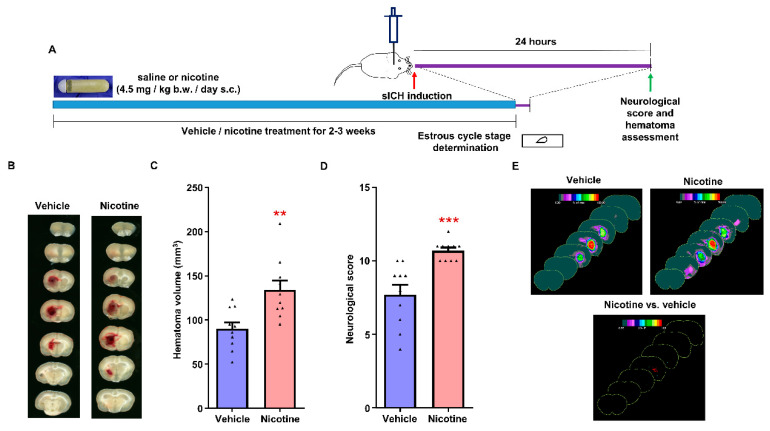
The effect of chronic nicotine treatment on hematoma expansion following collagenase-induced sICH in female rats: (**A**) experimental design; (**B**) representative example images showing hematoma volume following sICH; (**C**) hematoma volume; (**D**) neurological impairment; and (**E**) hematoma frequency maps at 7 coronal levels (bregma +6 mm to −6 mm). ** *p* < 0.01 and *** *p* < 0.001 vs vehicle group.

To determine the effect of chronic nicotine exposure on hematoma growth following sICH in male and female rats, rats were divided into the following treatment groups: (I) vehicle-treated male rats (*n* = 10); (II) nicotine-treated male rats (*n* = 10); (III) vehicle-treated female rats (*n* = 10); and (IV) nicotine-treated female rats (*n* = 10) (Figure 1A and Figure 2A).

The osmotic pump delivering the vehicle or nicotine to each animal was removed just before the sICH-induction surgery to mimic the clinical situation when sICH patients may not be able to smoke early during post-sICH hospitalization. 

### 2.9. Collagenase Activity Assay

The 2-furanacryloyl-l-leucylglycyl-l-prolyl-l-alanine (FALGPA) (Sigma-Aldrich, St. Louis, MO, USA) assay was employed to determine collagenase activity as described previously [31]. FALGPA solution was prepared in an assay buffer containing 50 mM tricine, 10 mM CaCl_2_, and 400 mM NaCl, pH 7.5. FALGPA solution (140 µL, 1.0 mM) and 5 µL nicotine (or saline) were mixed for the assay. The reaction was started by adding 5 µL (2.125 units) of collagenase (Type IV isolated from Clostridium histolyticum, Sigma-Aldrich, St. Louis, MO, USA) solution. The absorbance was measured at 345 nm for 10 min at 28 s intervals, and the maximum linear rate of decrease in absorbance (ΔA345/min) served as a measure of collagenase activity. Two nicotine concentrations (37.5 and 75 ng/mL) were used in the assays. Studies have shown that blood nicotine levels in subjects who smoke ranged between 6 to 215 ng/mL, and rat blood levels treated with a 4.5 mg/kg b.w. per day dose of nicotine was around 66 ng/mL [19,32]. A study showed that [33] plasma nicotine levels range from 20 to 50 ng/mL in subjects who smoke. Therefore, to mimic nicotine levels in smokers and nicotine-treated rodents, we selected 37.5 and 75 ng/mL nicotine concentrations to test the potential effect of nicotine on collagenase activity.

### 2.10. Statistical Analysis

Data were analyzed with GraphPad Prism software, version 5. Grubbs’ test was performed so that no outlier data points were included in the analysis (none detected). Two groups were compared using Student’s *t*-test. Two-way ANOVA was used to analyze the potential impacts of treatment and sex on physiological parameters and sICH outcomes. A value of *p* < 0.05 was considered statistically significant, and the results are expressed as mean ± SEM.

The following predefined criteria for exclusion were used in the study: (1) rats with a body weight above 375 g (none); (2) rats with a mean MABP for 10 min above 135 or below 80 mmHg before and after collagenase injection (none); (3) leaky or blocked syringe needle used for the collagenase injection (1 nicotine-treated female rat); (4) rats that did not receive the collagenase injection at the intended site in the brain (1 vehicle-treated male and female rat); (5) rats with blood glucose levels above 160 mg/dL (3 vehicle- and 2 nicotine-treated male rats; 1 nicotine-treated female rat); (6) rats with surgical complications such as inflammation at the site of osmotic pump implantation, severe blood loss or blockade in the vein or artery catheter during sICH surgery (1 vehicle-treated female rat); and (7) rats with blood gases outside the normal range (1 vehicle-treated female rat). Animal(s) that met any of these criteria were excluded from the study and replaced with additional animal(s). 

## 3. Results

### 3.1. Physiological Parameters

Physiological parameters were maintained within a normal range during the surgical procedure (Supplementary Table S1). A slight but significantly higher body temperature and blood pCO_2_ were observed in the nicotine-treated female group after collagenase injection when compared to the vehicle-treated group (Supplementary Table S1). However, we did not observe any substantial variations in any other physiological parameters among the study groups. Therefore, minor but significant changes observed in some of the measures are not expected to make any considerable impact on sICH outcome. 

We further analyzed the data to compare the effect of treatments and sex on physiological parameters using two-way ANOVA. Treatment significantly affected baseline values of body temperature (*p* < 0.01). Sex significantly affected baseline values of body weight (*p* < 0.001), body temperature (*p* < 0.05), blood pH (*p* < 0.001), pO_2_ (*p* < 0.05), glucose (*p* < 0.001) and MABP (*p* < 0.001). Sex significantly affected post-sICH values of blood pH (*p* < 0.001), pO_2_ (*p* < 0.001) and MABP (*p* < 0.01). Despite statistical significance, the extent of changes in most of the above physiological parameters was minor, and as expected, weights of female animals were lower than weights of male animals. The effect of treatment and sex was not statistically significant in terms of the remaining parameters measured at baseline and post-sICH. Therefore, we do not expect these slight variations in physiological parameters to affect sICH outcome.

### 3.2. The Effect of Chronic Nicotine Exposure on Hematoma Growth following sICH in Young Male Rats

We evaluated the effect of chronic nicotine exposure on hematoma volume following collagenase-induced sICH in young male rats. The male rats were treated with vehicle or nicotine for a period of 17 ± 2 or 16 ± 2 days, respectively. Total hematoma volume for the vehicle- and nicotine-treated young male groups was 98 ± 9 (*n* = 10) and 139 ± 9 mm^3^ (*n* = 10), respectively (Figure 1B,C). The hematoma volume was 42% (*p* < 0.01) higher in nicotine-treated male rats when compared to vehicle-treated rats. The hematoma frequency maps showed that the nicotine-treated male rats had larger hematomas at multiple coronal levels when compared to the vehicle-treated group (Figure 1E). The neurological score observed in the vehicle-treated group was 7.4 ± 0.6 (Figure 1D). However, the neurological score in the nicotine-treated group was significantly higher (9.3 ± 0.6, *p* < 0.05) than in the control group. These results show that chronic nicotine exposure enhances hematoma growth, as well as worsens neurological deficits post-sICH in male rats.

### 3.3. The Effect of Chronic Nicotine Exposure on Hematoma Growth following sICH in Female Rats

We evaluated the effect of chronic nicotine exposure on hematoma growth following collagenase-induced sICH in female rats. The female rats were treated with vehicle and nicotine for a period of 16 ± 1 and 18 ± 1 days, respectively. Total hematoma volume for the vehicle- and nicotine-treated female groups was 90 ± 7 (*n* = 10) and 134 ± 11 mm^3^ (*n* = 10), respectively (Figure 2B,C). 

The hematoma volume was 48% higher (*p* < 0.01) in the nicotine-treated female group when compared to the vehicle-treated group. Hematoma frequency maps also confirmed hematoma volume results (Figure 2E). The neurological score for the vehicle-treated group was 7.7 ± 0.7 (*n* = 10). However, the neurological score in the nicotine-treated group was significantly higher (10.7 ± 0.2, *p* < 0.001) (*n* = 10) than in the control group (Figure 2D). Therefore, our results demonstrate that chronic nicotine exposure increases hematoma growth in female rats following sICH.

### 3.4. The Difference of the Effect of Treatments on Outcome following sICH in Male and Female Rats

**Figure 3:**
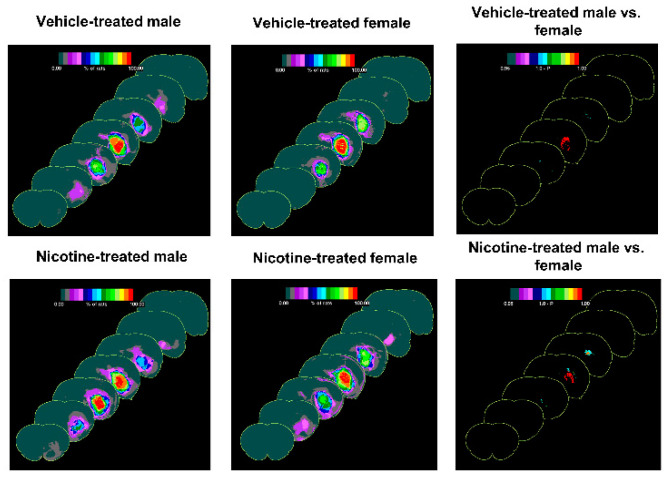
Hematoma frequency maps at 7 coronal levels (bregma +6 mm to −6 mm) showing the effect of chronic vehicle and nicotine treatment on hematoma expansion following collagenase-induced sICH in male and female rats.

We further analyzed the data to compare the effect of treatments and sex on hematoma volume and neurological score using two-way ANOVA. The analysis showed that while the effect of treatment was significantly different in terms of hematoma volume (*p* < 0.001) and neurological score data (*p* < 0.001), the effect of sex was not statistically significant in terms of hematoma volume and neurological score data. Hematoma frequency maps also confirmed the hematoma volume results (Figure 3). 

Although the core region of the hematoma in males appears denser than in the females, overall, the hematoma volume was not different between males and respective female groups.

### 3.5. The Effect of Nicotine on Collagenase Activity

**Figure 4:**
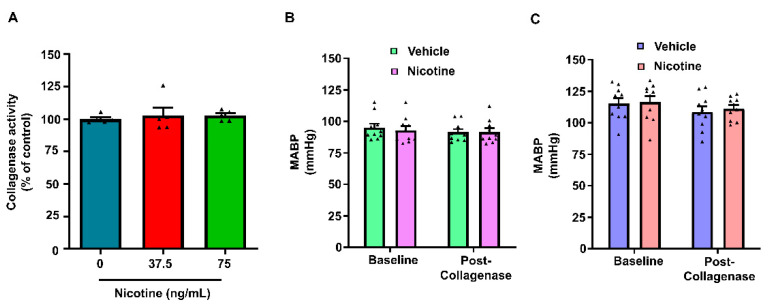
(**A**) The effect of nicotine on collagenase activity determined in vitro. Data are presented as percentage change in the rate of decrease in absorbance (ΔA345/minute) over control levels. Mean arterial blood pressure at baseline and following collagenase injection in (**B**) male and (**C**) female rats that received chronic vehicle or nicotine treatment in vivo.

To ascertain that collagenase activity is not affected in the presence of nicotine, we determined collagenase activity using an in vitro method in the presence of two different nicotine concentrations. Collagenase activity in the presence of 37.5 and 75 ng/mL nicotine was 103 ± 6% (*n* = 5) and 103 ± 2% (*n* = 5), respectively, when compared to collagenase activity in the absence of nicotine (100 ± 1%, *n* = 5) (Figure 4A). 

We did not find any statistically significant effect of nicotine on collagenase activity. These results demonstrate that the presence of nicotine in animals treated with nicotine will not have any impact on the activity of injected collagenase (Figure 4A). 

## 4. Discussion

Cigarette smoking is the second leading risk factor for mortality in the USA, and mortality in smokers is higher than that in non-smokers by a factor of 3 [10,34]. In 2018, approximately 15% of adults used e-cigarettes [35]. The use of electronic cigarettes is documented to increase the risk of myocardial infarction and stroke [36]. Potential ingredients that contribute to an adverse cardiovascular state with e-cigarette smoking include nicotine among others [37,38]. Tobacco use greatly enhances the risk of sICH, one of the most serious forms of stroke with high mortality and poor prognosis [10,12,39]. Further, smoking is shown to aggravate the outcome following sICH [12,16,17,40]. Animal models are ideal to test the effect of nicotine on sICH outcomes in a more controlled manner. Therefore, we determined the effect of chronic nicotine exposure on hematoma growth following sICH using a rat model. Our data in the present study show that nicotine exposure increases hematoma expansion and neurological impairment post-sICH in both male and female rats. 

Hematoma expansion occurs in human subjects during the early hours after the onset of sICH and, in some cases, continues until 1 day post-sICH [41,42,43]. An earlier study reported that current smoking correlates with post-sICH hematoma volume [40]. A previously published study [26] and unpublished data from our laboratory suggest that the rat model of collagenase-induced sICH is appropriate to study hematoma growth as hematoma following collagenase injection increases over time, similar to sICH patients. Therefore, we selected this model for studying the effect of nicotine exposure on hematoma expansion post-sICH. In the current study, we also confirmed that the presence of nicotine does not affect collagenase activity. Therefore, the observed effect of nicotine on hematoma growth is likely because of the chronic detrimental effects of nicotine on the brain and is unlikely to be a result of the potentiating effect of nicotine on collagenase activity. 

While cigarette smoking is known to activate platelets and the coagulation cascade, it also impairs fibrinolysis [44]. Wang et al. found that chronic nicotine exposure results in lower levels of tissue plasminogen activator in brain microvessels [21]. Therefore, it may be plausible that a slower hematoma resolution in the brain results in an increase in hematoma expansion and neurological deficits observed in chronically nicotine-treated rats. Additionally, as described in our earlier review article, other mechanisms such as nicotine-treatment-induced impaired blood–brain barrier permeability, increased reactive oxygen species production, and activation of pro-inflammatory pathways may also contribute to increased hematoma volume following sICH [12]. Controlled studies are needed to test such a hypothesis.

With an increase in hematoma volume, there is a substantial increase in sICH mortality [7,41], and mortality in sICH patients showing hematoma expansion (53.6%) is higher in comparison to patients who did not demonstrate hematoma expansion (6.3%) [45]. Therefore, hematoma volume is an important predictor of 30-day mortality [46]. In the present study, we observed that chronic nicotine exposure causes a substantial increase in post-sICH hematoma volume. This observation is in line with a clinical study, which showed an increased risk of hematoma expansion, related mortality, and worse outcomes following sICH in current smokers [47]. This study showed that with a 6 mL or 33% increase in hematoma volume, there is a significant increase in the chance of hematoma expansion among smokers versus non-smokers, particularly between 12 and 72 hours after sICH onset [47]. This underlines the significance of the detrimental effect of nicotine on the risk of post-sICH hematoma expansion. A clinical study has found that sICH among subjects with previous history of smoking is associated with poorer outcomes versus patients without smoking history, when measured in terms of the Glasgow coma scale [17]. Our current study shows that chronic nicotine treatment enhances bleeding in the brain and acute neurological deficits in rats post-sICH. However, we did not observe mortality in any of the study groups employed until the 24-hour time point when the animals were euthanized for hematoma assessment. Long-term studies are needed to evaluate the effect of nicotine exposure on post-sICH mortality and outcomes. 

Some studies have investigated the sex differences in outcomes following sICH. A previous study has shown that men are at a higher risk of death after one month from sICH than women [24]. Males have an increased chance of hematoma expansion in comparison to females [23]. Some studies have shown that in comparison to smoking men, the propensity for brain hemorrhage is higher in smoking women [48,49]. An earlier study demonstrated that the sICH severity is more profound in women in comparison to men [50]. Another study has, however, shown that the relative risk of hemorrhagic stroke is higher in smoking men than in smoking women [51]. Although future studies are required to confirm the risk of hemorrhagic stroke in smoking men versus smoking women, the published literature conclusively shows that tobacco use enhances the risk of hemorrhagic stroke in both sexes. In line with the above studies, our data show that chronic nicotine exposure increases hematoma growth in both male and female rats. Our data further show that the extent of the effect of nicotine exposure in male rats on hematoma expansion was similar to the extent observed in female rats. The difference between our conclusions about the relative effect of nicotine in rats of both sexes and previous clinical studies on the effect of tobacco exposure in human subjects might be attributed to possible ethnic and societal factors that come into play in the case of human subjects. Future studies remain to evaluate the long-term consequences of nicotine exposure on sICH in young and aged animals to understand the pathological features of the detrimental effect of nicotine on different subsets of sICH patients.

One study investigated the procoagulant effect of nicotine on sICH outcome [44]. Acute administration of nicotine (2 mg/kg b.w., i.p.) once daily for three consecutive days starting 3 hours after collagenase-induced sICH and cortical hemorrhage in mice improved motor performance and decreased cell death in the brain, but did not exert any effect on hematoma or lesion volume [52,53]. A meta-analysis of studies that evaluated the effect of nicotine administration in non-smokers and smokers showed that nicotine produces significant positive effects on motor abilities in humans [54]. Therefore, the beneficial effect of nicotine on motor performance of hemorrhagic rats in these studies may be due to its direct effect on behavior rather than due to improvement in hemorrhagic stroke. In comparison to the above-mentioned sICH study, we employed a higher dose and discontinued nicotine treatment (as mentioned above) prior to sICH induction. Acute and selective activation of α-7 nicotinic acetylcholine receptor increases surviving neurons [55] and improves functional outcomes following sICH [56]. The beneficial effect of α-7 nicotinic acetylcholine receptor activation 1 h prior to sICH was reduced when compared to the effect of receptor activation 3 h after sICH. Treatment with the activator of α4β2 nicotinic acetylcholine receptor did not result in any beneficial effect [55]. Nicotine is expected to simultaneously activate both the α-7 and α4β2 nicotinic acetylcholine receptors. The synergistic effect of nicotine-induced activation of α-7 and α4β2 nicotinic acetylcholine receptors in the brain on sICH outcome is not well understood. Treatment with neither α-7 nor α4β2 nicotinic acetylcholine receptor activators affects hemorrhage size [55]. These studies demonstrate that post-sICH activation of specific nicotinic acetylcholine receptors does provide neuroprotective effects without affecting hemorrhage size. In contrast, the observations reported here suggest that prior chronic exposure to nicotine results in worsened post-sICH outcomes. It may be plausible that the chronic presence of nicotine in the brain might potentiate detrimental effects on the brain, which result in poor outcome. Future studies are required to confirm the present findings using other models of hemorrhagic stroke. Further investigations are also warranted to understand the mechanism by which chronic nicotine exposure results in pathological changes in the brain in relation to worsened outcomes following sICH. It would also be of interest to study the potential involvement of weakening of blood vessel structure such as loss of blood–brain barrier integrity in mediating the observed effect of nicotine.

## 5. Conclusions

Overall, we conclude that chronic nicotine exposure not only enhances hematoma growth but also worsens neurological outcomes in male and female rats. Identifying the mechanism by which nicotine increases hematoma expansion will help us understand the effect of tobacco use on sICH outcomes in human subjects.

## Data Availability

Not applicable.

## Supplementary Materials

The following supporting information can be downloaded at: https://www.mdpi.com/article/10.3390/biom12050621/s1, Table S1: Physiological parameters before and after collagenase injection in rats.
