# Supplementary material for: Chronic Nicotine Exposure Increases Hematoma Expansion following Collagenase-Induced Intracerebral Hemorrhage in Rats
Source: Biomolecules. 2022 Apr 21;12(5):621. doi: 10.3390/biom12050621 (PMC9138464; doi:10.3390/biom12050621)
Supplement: Supplementary file 1 [file biomolecules-12-00621-s001.zip › biomolecules-1679151-supplementary.pdf]

SUPPLEMENTAL MATERIAL

Table S1. Physiological parameters before and after collagenase injection in rats.

Groups		Sampling time	Body Weight (g)	Body Temperature (°C)	Head Temperature (°C)	pH	pCO_2 (mmHg)	pO_2 (mmHg)	MABP (mmHg)	Blood glucose (mg / dL)
Male rats	Vehicle-treated (n=10)	Before injection	322 ± 9	37.1 ± 0.1	36.7 ± 0.1	7.39 ± 0.02	37 ± 1	109 ± 5	103 ± 2	114 ± 7
		After injection		37.0 ± 0.1	36.8 ± 0.1	7.39 ± 0.02	36 ± 0	114 ± 5	101 ± 2	
	Nicotine-treated (n=10)	Before injection	341 ± 10	37.0 ± 0.1	36.7 ± 0.0	7.39 ± 0.02	37 ± 1	107 ± 7	99 ± 2	112 ± 8
		After injection		37.1 ± 0.1	36.7 ± 0.1	7.39 ± 0.02	35 ± 1	124 ± 6	100 ± 3	
Female rats	Vehicle-treated (n=10)	Before injection	261 ± 5	37.1 ± 0.1	36.6 ± 0.0	7.32 ± 0.01	37 ± 1	92 ± 4	109 ± 2	144 ± 5
		After injection		36.8 ± 0.1	36.7 ± 0.1	7.32 ± 0.01	35 ± 1	95 ± 4	106 ± 2	
	Nicotine-treated (n=10)	Before injection	266 ± 6	36.8 ± 0.1	36.5 ± 0.0	7.31 ± 0.01	39 ± 1	99 ± 5	112 ± 3	139 ± 7
		After injection		37.0 ± 0.1*	36.8 ± 0.1	7.32 ± 0.01	36 ± 1**	103 ± 3	110 ± 3	

* $p<0.05$ vs respective control.
** $p<0.01$ vs respective control.
